# Diabetic health literacy and associated factors among diabetes mellitus patients on follow up at public hospitals, Bale Zone, South East Ethiopia, 2021

**DOI:** 10.1371/journal.pone.0270161

**Published:** 2022-07-07

**Authors:** Hailye Mamo Mogessie, Mulugeta Adugnew Gebeyehu, Mitaw Girma Kenbaw, Tesfaye Assefa Tadesse

**Affiliations:** 1 Department of Nursing, School of Health Science, Goba Referral Hospital, Madda Walabu University, Goba, Ethiopia; 2 Department of Nursing, School of Health Science, Wollo University, Dessie, Ethiopia; Indiana University Purdue University at Indianapolis, UNITED STATES

## Abstract

**Objective:**

This study was aimed to assess diabetic health literacy and associated factors among adult diabetic patients in public hospitals, Bale Zone, Southeast Ethiopia.

**Methods:**

A hospital-based cross-sectional study was conducted among 402 diabetic patients in three public hospitals and the samples were selected using simple random sampling technique. The comprehensive functional, communicative, and critical health literacy questionnaire was used to measure diabetic health literacy. Descriptive statistics and Ordinary logistic regression analyses were conducted, and a P-value of < 0.05 was considered to declare a result as statistically significant.

**Result:**

A total of 402 diabetic patients were involved in the study. Of all respondents, 41.8%, 27.9%, and 30.3% had low, moderate, and high diabetic health literacy respectively. Educational status; can’t read and write (AOR = 0.085;95% CI: 0.03,0.26), can read and write (AOR = 0.10; 95% CI: 0.04,0.30), primary school (AOR = 0.25; 95% CI: 0.09,0.67), secondary school (AOR = 0.37; 95% CI: 0.14,0.99), duration of onset ≤5 years (AOR = 2.05; 95% CI:1.09,4.19), being not member of DM association (AOR = 0.43; 95% CI: 0.26,0.73), having ≤ 3 diabetes mellitus information sources (AOR = 0.15; 95% CI: 0.03,0.77), social support; poor (AOR = 0.40;95% CI: 0.21,0.79), and moderate (AOR = 0.50; 95% CI: 0.28,0.92) were significantly associated with diabetic health literacy.

**Conclusion:**

A substantial number of diabetic patients had low diabetic health literacy. Educational status, Sources of diabetic information, Member of DM association, and social support were significantly and positively associated with diabetic health literacy. But, duration of diabetes onset was negatively associated with diabetic health literacy of respondents. So, we recommend providing readable and picturized printed materials, and diabetic patient education to be considered.

## Introduction

Diabetes mellitus (DM) is a global public health problem that occurs when there are raised levels of glucose in a people’s blood because their pancreas either cannot produce any or enough of the hormone insulin or cannot effectively use the insulin it produces [1]. Diabetes is an increasing health problem internationally with a global prevalence of 9.3% [2]. According to International Diabetes Federation report in 2019, the number of diabetic patients will rise to 700 million by 2045 [3]. In sub-Saharan Africa (SSA), DM is predicted to increase quickly, rising by 80% over 20 years, and will affect around 18.7 million Africans by the year 2025 [4].

Diabetes is not fatal if managed effectively, but untreated hyperglycemia results in various multi-organ complications that cause acute and chronic morbidity including end-stage renal disease, blindness, amputations, and finally death. To prevent diabetic complications patients are expected to know about the disease, self-management, and self-care abilities [5]. One of the factors influencing the level of knowledge and control of the disease is the level of diabetic health literacy of the patients [6].

World Health Organization (WHO) has identified health literacy as one of the greatest determinants of health, which defines it as “the cognitive and social capabilities that determine the motivation and ability of people to gain access, understand, and utilize information in such ways that enhance and maintain good health” [1]. Diabetic health literacy is the degree to which patients with diabetes have the necessary skills and abilities to gain, understand, analyze, communicate, and enumerate diabetes-related information both in the healthcare settings and daily lives for treating and self-managing their health condition [7].

Even though there are many advancements in diabetes mellitus prevention, treatment, and disease control on the occurrence of diabetic complications (end-stage renal disease, blindness, and amputations) was still high [8, 9]. However, many researchers suggest that diabetes control and care need the empowerment of patients in self-care behaviors [10]. The effective factors in managing diabetes include the provision of education and training, adequacy of disease knowledge, and proper diabetes control. One of the influencing factors playing a major role in the level of knowledge and disease control is health literacy [11].

Diabetic health literacy is a major obstacle in diabetes treatment and an important nonclinical factor [12]. Study shows that patients with low health literacy faces some challenges in realizing and accessing health-related information, and are getting difficulties in expressing their status to health care providers, resulting in poor self-management [13]. Diabetic patients with low diabetic health literacy levels are subjected to more hospitalizations, poorer preventive behaviors, more medical expenses, and poor glycemic control which leads to several complications [14]. In contrast, patients with high levels of diabetic health literacy are more likely to take part in health-promoting behaviors; therefore, they have better health outcomes [12].

A recent systematic review, conducted in seven countries showed that the prevalence of limited diabetic health literacy ranged from 7.3% to 82% [15]. In Ethiopia, the prevalence of high diabetic health literacy was 56.5%% [13]. Regarding the factors, different studies across the globe revealed that age especially older ages, educational status, occupational type, place of residence, marital status, duration of the disease, treatment regimen, comorbidity, hospitalization history, and family history were associated with DHL of the patients.

In addition, to this, the factors associated with diabetic health literacy were not assessed in our country. Due to these facts, the assessment of diabetic health literacy and the associated factors was found to be advantageous to fill the gap in knowledge, design programs, strategies for effective health education, and patient empowerment. In conclusion, this study was aimed to assess the magnitude of health literacy and associated factors among adult diabetic patient outpatient clinics in public health hospitals of Bale Zone, 2021.

## Methods

### Study design, setting, and period

An institution-based cross-sectional study was conducted at three public hospitals namely Robe general hospital (RGH), Delo Mena general hospital (DGH), and Madda Walabu University Goba Referral hospital (MWU GRH) found in Bale zone. The zone is located 430 km southeast of the capital city Addis Ababa, Ethiopia. The zone had a total population of 1,965,937 in 2019 and 18 woreda and two towns. There are five hospitals, 82 health centers, and 381 health posts in this zone. The monthly flow of diabetic patients who had follow up in the selected three hospitals were 1236. Of these patients, 52 were attending their follow up in RGH, 911 in MWU GRH, and 273 were in DGH. The study was conducted from March 26 to April 25, 2021.

### Study population

The study population was all adult diabetic patients from diabetic follow-up clinics during the study period at Bale Zone public hospitals (Robe, MWUGRH, and Delomena hospitals).

### Inclusion and exclusion criteria

All adult diabetic patients who attend their follow ups for one and more than one year, whose age was ≥ 18 years old, and patients who were available during the data collection period and volunteered to participate, were included in the study. While patients with gestational diabetes, severely ill patients, and mentally ill patients with cognitive impairments, were excluded.

### Sample size determination and sampling technique

The sample size was determined using a formula for single population proportion formula

$$\text{n}={\left(\text{Z}\alpha_{/2}\right)}^{2}\;\text{p}\left(1-\text{p}\right)/{\text{d}}^{2}$$

Where; n = Minimum sample size for a statistically significant survey

z = is the significance level (at 5% significance level its value is 1.96)

p = is the proportion of adequate health literacy among diabetic patients.

d = is the margin of error (It has been taken as 5%).

Since there was a previous study conducted on diabetic health literacy, the proportion of diabetic patients having high diabetic health literacy was 56.5% [13]. SO, the sample size was calculated as follows:

$$\begin{array}{l} \text{n}={\left(1.96\right)}^{2}\times \left(0.565\right)\times \left(0.435\right)/{\left(0.5\right)}^{2}\\ \text{n}=378. \end{array}$$

The total numbers of diabetic patients attending the outpatient clinics of the selected three hospitals were 1236 patients. Therefore, considering a 10% non-response rate, the final sample size was 416.

**Fig 1:**
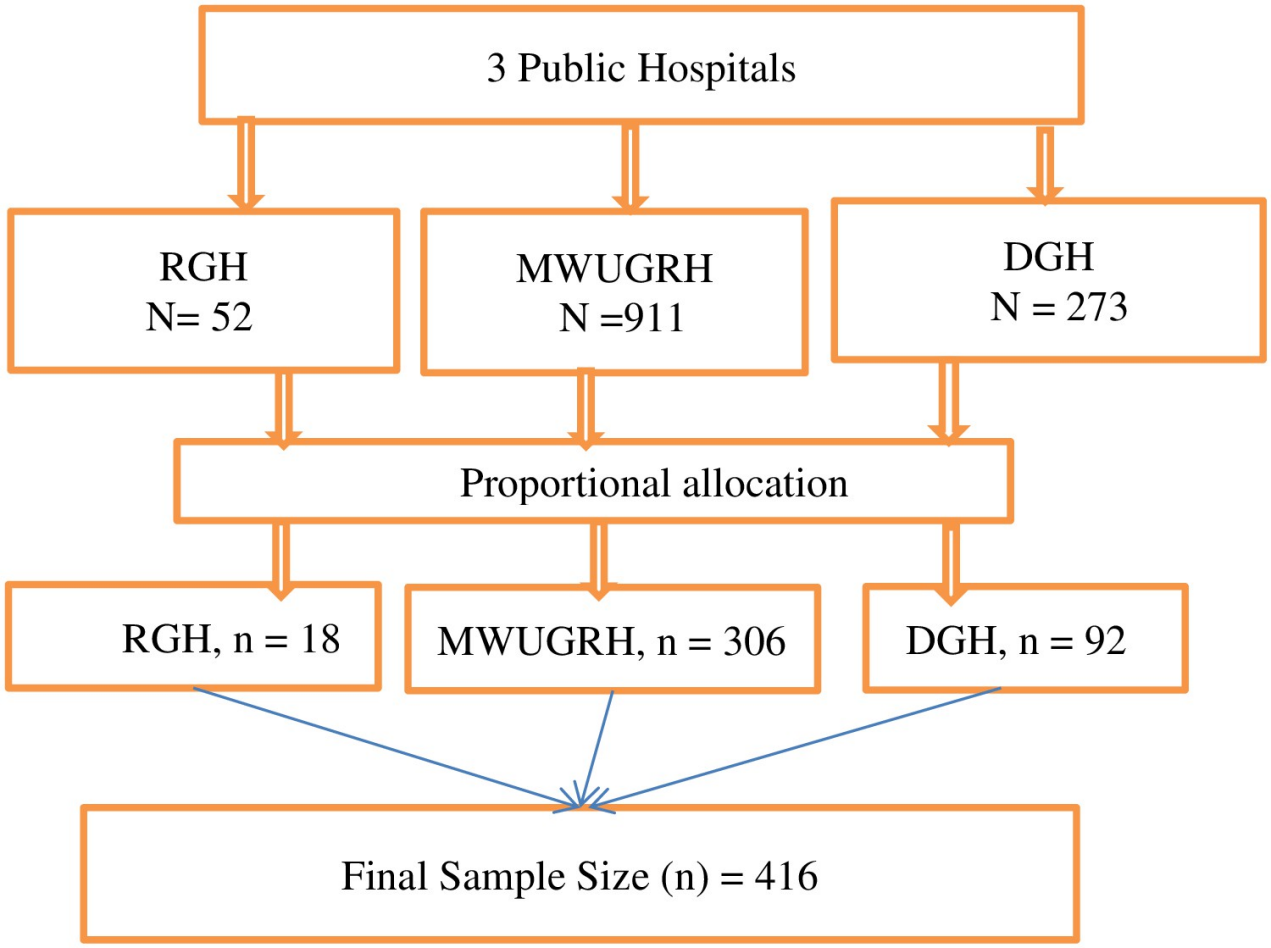
Schematic presentation of sampling technique used to select the study subjects from public hospitals in Bale Zone, South East Ethiopia, 2021.

The simple random sampling technique was used to select study participants. When the patients came for follow up, their medical record cards were reviewed for the inclusion criteria. The number of study participants from the selected public hospitals was determined from the current total number of diabetic patients who have follow up which is 1236 in three hospitals. Samples were allocated to each of the selected Hospitals based on proportional allocation to sample size. The lists of respondents or sampling frames were obtained from the updated registration books of each follow-up clinic of the hospitals. After establishing the sampling frames of respondents, simple random sampling technique was used to identify the study unit to be included in the study. The diabetic patient who met the inclusion criteria were selected until the required sample size was achieved (Fig 1).

### Data collection procedure

Data was collected by six trained nurses using a structured pretested and validated questionnaire which was contextualized to the study area through face-to-face interviews. The principal investigator and an assistant were continuously supervised the data collection. It was prepared originally in English and was translated to Amharic and Afan Oromo and then translated back to English for checking the consistency of the questionnaire. It consisted of (1) socio-demographic characteristics; (2) diabetic related clinical information; (3) Patients Social Support which was measured by the Oslo Social Support Scale (OSSS-3). The total score was calculated by adding up the raw scores for each item. The sum of the raw scores ranges from 3 to 14. A score of 3–8 was classified as poor support, a score of 9–11 as moderate support, and a score of ≥12 strong support [16]. (4) Diabetic health literacy which was measured by 14 item FCCHL questions with 5 point Likert scales [17]. The mean score was calculated and switched to the percentage (5 points as 100%) used to determine the level of diabetic health literacy in which scores of ≥ 75, 60–74, and ≤ 59 were regarded as high, medium, and low diabetic health literacy respectively [13]. The pretest was conducted in Dodola general hospital on 21 diabetic patients. After the pretest reliability test was done to confirm its consistency and also we made some amendments and rearrangements to questions.

### Data entry and statistical analysis

The collected data was checked for its completeness. Then, data was coded, entered, and cleaned using Epi Data version 3.3.1 software and finally was exported into SPSS version 25.0 software for analysis. A reliability test was performed to check the consistency of the diabetic health literacy questions (Cronbach’s α = 0.93). Summary statistics were done for the outcome and independent variables. Ordinary logistic regression analysis was employed, in which bi-variate logistic regression analysis was carried out and variables with a p-value less than 0.25 in this analysis were included in the multivariate logistic regression analysis, and finally, significant factors were identified based on 95% confidence level adjusted odds ratio (AOR) and p-value ≤0.05. The test of parallel lines did not bear any significant difference (p = 0.72), indicating no violation of the proportional odds assumption, and the ordinal regression analysis was the best fitted model for predicting the associations.

### Ethical approval and consent to participate

This study was approved by the Ethical committee of Madda Walabu University. Besides, an official letter was issued from the school of Health Sciences, Goba Referral Hospital to the director of each hospital. After explaining the purpose of the study, written informed consent was obtained from each of the study participants. All information collected from the participants was kept confidential.

## Results

### Socio-demographic characteristics of the respondents

Four hundred two adult diabetic patients were participated in the study. 250 (62.2%) of the respondents were male, 41.55 ± 14.96 was the mean age, 276(68.7%) were married, 94(23.4%) were unable to read and write, and 311(77.4%) of respondents did not use internet to search diabetic related information (Table 1).

**Table 1:** Socio-demographic characteristics of diabetic patients at the outpatient clinic of public hospitals, Bale Zone, 2021(n = 402).

Variables		Frequency n(%)
Sex	Male	250(62.2%)
	Female	152(37.8%)
Age, in years	< 40 years	204(50.7%)
	40–60 years	140(34.8%)
	>60 years	58(14.4%)
Marital status	Single	76(18.9%)
	Married	276(68.7%)
	Divorced	23(5.7%)
	Widowed	27(6.7%)
Educational level	Unable to read and write	94(23.4%)
	Able to read and write	83(20.6%)
	Primary school	90(22.4%)
	Secondary school	78(19.4%)
	College graduate & above	57(14.2%)
Occupation	Student	56(13.9%)
	Self-employed	199(49.5%)
	Employed	53(13.2%)
	Unemployed	25(6.2%)
	Housewife	61(15.2%)
	Others	8(2.0%)
Place of residence	Urban	249(61.9%)
	Rural	153(38.1%)
Monthly income	< 2000 ETB	99(24.6%)
	2001–5000 ETB	199(61.9%)
	> 5000 ETB	104(25.9%)
DM information source	≤ 3 sources	374(93%)
	> 3 sources	28(7%)
Internet use	Yes	91(26.6%)
	No	311(77.4%)
Smoking	Yes	24(6.0%)
	No	378(94%)
Alcohol	Yes	44(10.9%)
	No	358(89.1%)

### Diabetic related clinical information of the respondents

In this study, 252(62.7%) of the respondents were T2 DM, 140(34.8%) had a family history of diabetes, 204(50.7%) were on insulin injection, and 214(53.2%) did not receive diabetic education. The majority of the respondents 248(61.7%) were not a member of diabetic association (Table 2).

**Table 2:** Diabetes related clinical information of diabetic patients at the outpatient clinic of public hospitals, Bale Zone, 2021(n = 402).

Variables	Categories	Frequency n(%)
Type of DM	T1 DM	150(37.3%)
	T2 DM	252(62.7%)
Family history	Yes	140(34.8%)
	No	262(65.2%)
Duration of diabetes	‘1–5’ years	221(55%)
	‘6–10’ years	116(28.9%)
	> 10 years	65(16.2%)
Treatment regimen	Insulin injection	204(50.7%)
	Oral hypoglycemic agent	162(40.3%)
	Both insulin & OHA	36(9%)
Diabetic education received	Yes	188(46.8%)
	No	214(53.2%)
Member of diabetic association	Yes	154(38.3%)
	No	248(61.7%)
Comorbidity	Yes	142(35.3%)
	No	260(64.7%)
History of admission	Yes	262(65.2%)
	No	140(34.8%)

### Level of social support of diabetic patients

Among the respondents, 224(55.7%) have poor social support. The majority 147(36.6%) of the respondents had ’1–2’ people so close to them to support if they faced a great personal problem, one fourth 101(25.1%) had no support, most 113(28.1%) of the respondents have difficulty to get practical help from a neighbor, and 99(24.6%) can easily get practical help from a neighbor (Table 3).

**Table 3:** Level of social support of diabetic patients at the outpatient clinic of public hospitals, Bale Zone, 2021(n = 402).

Level of social support	Frequency	Percentage (%)
Poor social support	224	55.7%
Moderate social support	103	25.6%
Strong social support	75	18.5%

### Diabetic health literacy of the respondents

**Fig 2:**
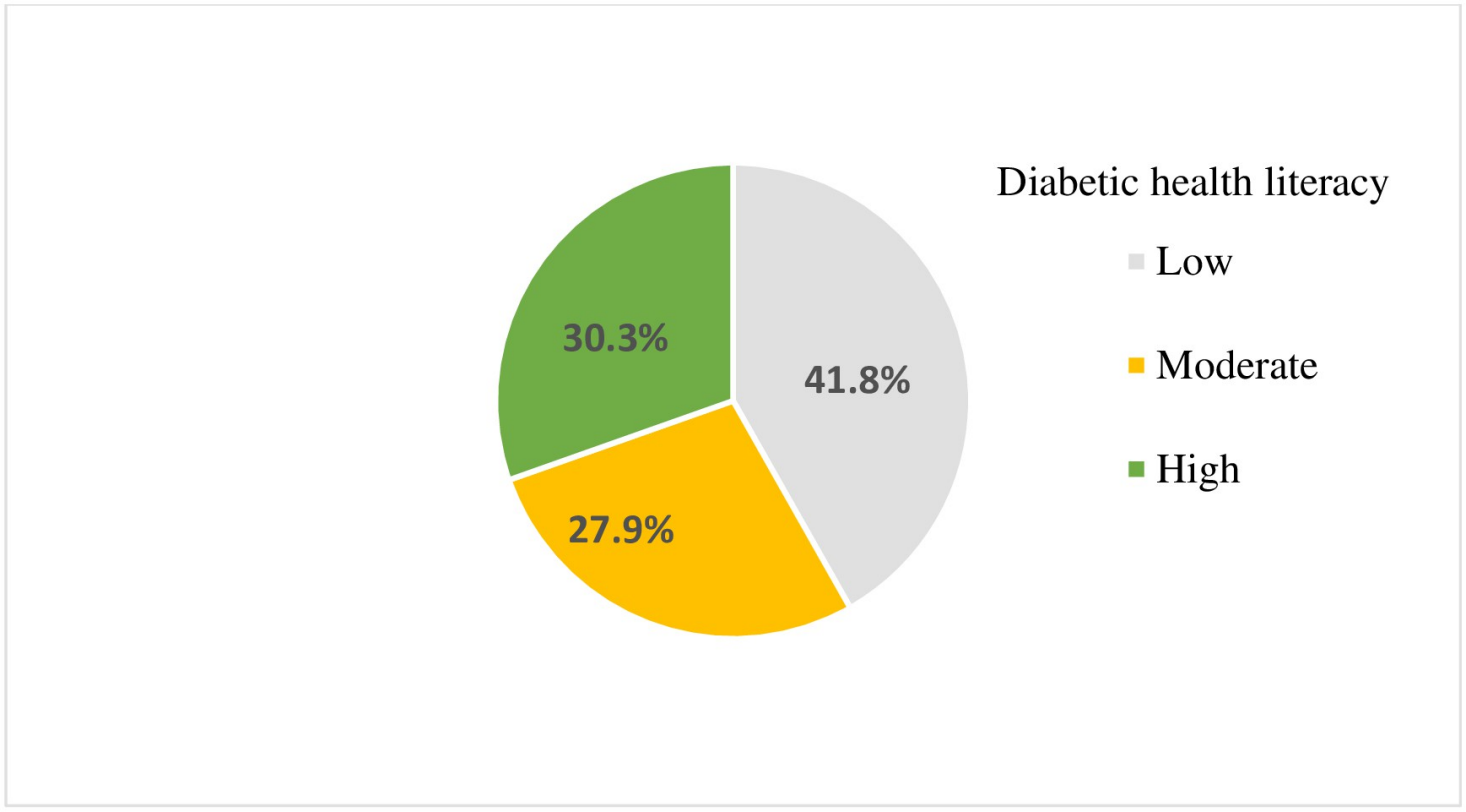
Magnitude of diabetic health literacy of diabetic patients at the outpatient clinic of public hospitals, Bale Zone, 2021(n = 402).

The mean diabetic health literacy score was 3.08 ± 0.901. One hundred sixty-eight (41.8%) of the respondents had poor diabetic health literacy (Fig 2).

More than half (n = 210, 52.2%) of respondents either agree or strongly agree that they find characters that they cannot read. 105(26.1%), and 96(23.9%) of respondents strongly agreed and agree that they need someone to help them to read hospital or pharmacy instructions or information leaflets respectively. In the question that assesses, the status of collecting information to make their health care decisions was agree (24.4%) and strongly agree (8.5%) (Table 4).

**Table 4:** Diabetic health literacy of diabetic patients at the outpatient clinic of public health hospitals, Bale Zone, 2021(n = 402).

Diabetic health literacy questions	Strongly Disagree	Disagree	Neutral	Agree	Strongly Agree
You find characters that you cannot read	57(14.2)	80(19.9)	55(13.7)	129(32.1)	81(20.1)
You find that the print was too small to read (even if you wear glass)	63(15.7)	78(19.4)	69(17.2)	120(29.9)	72(17.9)
You feel the content was too difficult for you to understand	64(15.9)	77(19.2)	75(18.7)	120(29.9)	66(16.4)
It takes to you a long time to read them	68(16.9)	67(16.7)	81(20.1)	109(27.1)	77(19.2)
you need someone to help you to read them	86(21.4)	64(15.9)	51(12.7)	96(23.9)	105(26)
You collect information from various sources	41(10.2)	112(27.9)	55(13.7)	155(38.6)	39(9.7)
You extract the information you wanted	54(13.4)	118(29.4)	118(29.4	86(21.4)	26(6.5)
You understand the information that you obtained	41(10.2)	99(24.6)	97(24.1)	138(34.3)	27(6.7)
You tell your opinion about your illness to your doctors, families, or friends	24(6)	74(18.4)	78(19.4)	194(48.3)	32(8)
you apply the obtained information to your daily life	27(6.7)	89(22.1)	95(23.6)	139(34.6)	52(12.9)
You consider whether the information is applicable to you or not	28(7)	122(30.3)	90(22.4)	139(34.6)	23(5.7)
You consider whether the information is credible	28(7)	126(31.3)	114(28.4)	113(28.1)	21(5.2)
You checked weather the information is valid and reliable	36(9)	117(29.1)	125(31.1)	104(25.9)	20(5)

### Factors associated with diabetic health literacy of respondents

In our study, keeping other variables constant, the odds of being in combined categories of moderate and high DHL is 92% less likely for those who cannot read and write (P ≤ 0.001, AOR = 0.085; 95%CI (0.028, 0.256); can read and write 90% (P ≤ 0.001, AOR = 0.10; 95% CI (0.036, 0.301); primary school 75% (P = 0.006, AOR = 0.25; 95%CI(0.090,0.674)) and secondary school 67% (P = 0.049, AOR = 0.37; 95%CI (0.137,0.994)) compared to those who are college graduate and above.

The odds of being in combined categories of moderate and high DHL is 2 times more likely for diabetic patients with 1–5 years duration of disease compared to those who had 10 and above years of diabetic disease duration (P = 0.048, AOR = 2; 95%CI (1.09,4.19)). The odds of being in combined categories of moderate and high DHL is 57% less likely for those who are not member of diabetic association as compared to patients who are member of diabetic association; (P = 0.002, AOR = 0.43; 95% CI (0.255, 0.725).

The odds of being in combined categories of moderate and high DHL is 85% less likely for those who had ≤ 3 sources of diabetic information compared to the group having > 3 DM information sources; (P = 0.023, AOR = 0.15; 95% CI (0.029,0.767)). The odds of being in combined categories of moderate and high DHL versus low DHL is 60% less likely for patients who had poor social support (P = 0.008, AOR = 0.40; 95% CI (0.21,0.79)), and 50% less likelihood for patients who had moderate social support (P = 0.025, AOR = 0.50; 95% CI(0.29,0.92 (Table 5).

**Table 5:** Ordinary logistic regression analysis of factors associated with diabetic health literacy of respondents.

Variables	Diabetic health literacy			COR (95%CI)	AOR(95%CI)	*P-value*
	Low	Moderate	High			
**Educational status**						
Can’t read & write	70(74.5)	15(15.9)	9(9.6)	0.03(-1.77,0.05)	0.085(0.028,0.256)	**≤0.001** *
Can read & write	52(62.7)	16(19.3)	15(18)	0.05(0.02,0.09)	0.104(0.036,0.301)	**≤0.001** *
Primary school	31(34.4)	36(40)	23(25.6)	0.12(0.06,0.25	0.246(0.090,0.674)	**0.006** *
Secondary school	15(19.2)	30(38.5)	33(42.3)	0.25(0.12,0.53)	0.369(0.137,0.994)	**0.049** *
College graduate & above	0(0)	15(26.3)	42(73.7)	1	1	-
**Duration of the disease**						
1–5 years	84(38)	68(30.8)	69(31.2)	1.56(1.01,2.71)	2.05(1.09,4.19)	**0.048** *
6–10 years	49(42.2)	33(28.4)	34(29.4)	1.36(0.77,2.41)	1.42(0.678,2.986)	0.351
> 10 years	35(53.8)	11(16.9)	19(29.3)	1	1	-
**Membership of DM association**						
No	111(44.8	68(27.4)	69(27.8)	0.45(0.31,0.87)	0.43(0.255,0.725)	**0.002** *
Yes	57(37)	44(28.6)	53(34.4)	1	1	-
**Source of DM information**						
≤ 3 sources	168(44.9	110(29.4)	96(25.7)	0.26(0.07,0.94)	0.15(0.029,0.767)	**0.023** *
> 3 sources	0(0)	2(7.1)	26(92.9)	1	1	-
**Social support**						
Poor	123(54.9	67(29.9)	34(15.2)	0.35(0.21,0.57)	0.40(0.21,0.79)	**0.008** *
Moderate	22(21.4)	21(20.4)	60(58.2)	0.37(0.24,0.61)	0.50(0.28,0.92)	**0.025** *
Strong	23(30.7)	24(32)	28(37.3)	1	1	-

CI-Confidence Interval:

*P ≤ 0.05—statistically significant value;

AOR—Adjusted Odds Ratio COR-Crude Odd Ratio

## Discussion

The current study was conducted to assess the magnitude of diabetic health literacy and associated factors among adult diabetic patients in public hospitals, Bale Zone, Ethiopia. The study ascertained that high diabetic health literacy among the respondents was 30.3% (95% CI 25.4, 34.8). This finding was higher than the study conducted on the urban population of Bangladesh (24%) [18], Jamaica (13.6%) [2], Iran(18.2%) [19], and Rwanda (14.3%) [4] respectively. This discrepancy might be due to variations in the tools utilized, socio-cultural and geographical differences. Additionally, it might be due to differences in the healthcare delivery system.

On the contrary, our finding was lower than the study conducted in Gondar, Ethiopia (56.5%). This might be due to the difference in tools utilized and the educational status of respondents. In this study, the percentage of educational status of participants who cannot read and write and can read and write was higher (44%) than revealed in Gondar (38.3%). This might leads to the difference that respondents who either agree or strongly agree with the questions; who can read and understand educational materials and leaflets were 52.2%, explain their diabetic conditions to health care providers (56.3%), and understand diabetic related medical information(41%) in our study and which was 64.1%, 79.3%, and 64.8% in the study conducted in Gondar [13].

This study revealed that educational status was significantly associated with the diabetic health literacy of the respondents. This finding was in agreement with a study done in China, higher educational attainment is associated with high diabetic health literacy [14], and in Gonder, where the mean score is higher for those attending higher education [13]. This might be due to respondents who joined college and universities had a better chance to learn from courses and social media than those who were not joined.

This study showed that the odds of being in combined categories of moderate and high DHL is 2 times more likely for diabetic patients with 1–5 years duration of disease compared to those who had 10 and above years of diabetic disease duration (P = 0.048, AOR = 2; 95%CI (1.09,4.19)). This finding is in agreement with the study conducted by [14] in china, who also found that patients who had lived less than years with diabetes had high diabetic health literacy, and on the contrary, those who lived more years with diabetes had low diabetic health literacy.

Our study ascertained that the odds of being in combined categories of moderate and high DHL is 57% less likely for those who are not member of diabetic association as compared to patients who are member of diabetic association; (P = 0.002, AOR = 0.43; 95% CI (0.255, 0.725). This might be due to the organization providing discussion meetings to access diabetic related information, ways of preventive measures, and treatment opportunities for patients. This also might be due to the majority of the respondents having primary education and above being member of DM association in this study, and education had a direct association with diabetic health literacy. So, they can grasp valuable information regarding DM.

This study also revealed that exposure to diabetic related information sources was found to be positively associated with diabetic health literacy of diabetic patients. Respondents with less than or equal to three information sources about DM were 85% less likely to have high diabetic health literacy. Among the study participants, the majority (97.3%) were using health care professionals as an information source, and the result of another study conducted in Debre Markos showed that a higher number (88.6%) of the study participants reported that their source of information were health care professionals [20]. This might be for the reason that health care professionals are easily accessible to patients with diabetes mellitus as compared to the other informational sources.

This study, also ascertained that social support was associated with the diabetic health literacy of respondents. This finding was consistent with a study conducted in China, which stated that patients having more social support had more likely to have better diabetic health literacy [14]. This might be due to the reason that individuals who had family and social relatives could have better information related to disease, have a chance to get an education, and may have got a chance to share their sources of information.

## Conclusion

Generally, our study revealed that a substantial number of adult diabetic patients had low diabetic health literacy, which is very important to controlling diabetes mellitus. Providing readable printed materials like leaflets and books regarding diabetes, and providing diabetic patient education and picturized printed materials to patients should be considered. Moreover, educational status, sources of diabetic information, members of DM association, and social support were significantly and positively associated with the diabetic health literacy of DM patients. But, the duration of diabetes onset was negatively associated with the diabetic health literacy of respondents.

### Limitation

Since the data on diabetic health literacy was collected through self-reporting rather than direct observation, social desirability bias, and the possibility of recall biases were limitations of this study.

## Supporting information

S1 File English version questionnaire. (PDF)

S2 File Gaaffii afaan oromoo. (PDF)

S3 File (ZIP)

## Abbreviations

ADA: American Diabetes Association
CI: Confidence Interval
DGH: Delomena General Hospital
DHL: Diabetic Health Literacy
ETB: Ethiopian Birr
IDF: International Diabetes Federation
MWUGRH: Madda Walabu University Goba Referral Hospital
RGH: Robe General Hospital
SD: Standard Deviation
SPSS: Statistical Package for Social Science Research
T1 DM: Type 1 Diabetes Mellitus
T2 DM: Type 2 Diabetes Mellitus
WHO: World Health Organization
