# Supplementary material for: Diabetic health literacy and associated factors among diabetes mellitus patients on follow up at public hospitals, Bale Zone, South East Ethiopia, 2021
Source: PLoS One. 2022 Jul 7;17(7):e0270161. doi: 10.1371/journal.pone.0270161 (PMC9262198; doi:10.1371/journal.pone.0270161)
Supplement: S1 File — (PDF) [file pone.0270161.s001.pdf]

English Version Questionnaire

Questionaires on Diabetic health literacy and associated factors for diabetes patients

Part I: - Socio demographic related information of patients

Instruction: These are questions regarding socio-demographic information of participants, please encircle the answer they responded to space provided.

No.	Variable	Response	Skip to
101	Age	…….yrs.	
102	Sex	1. Male<br>2. Female	
103	Marital status	1. Single<br>2. Married<br>3. Divorced<br>4. Widowed	
104	What is the highest educational level you completed?	1. Unable to read and write<br>2. Able to read and write<br>3. Elementary school<br>4. High school<br>5. Higher institution	
105	What is your current occupation?	1. Student<br>2. Self employed<br>3. Employed<br>4. Unemployed<br>5. House wife<br>6. Other specify……….	
106	Place of residence	1. Urban<br>2. Rural	
107	How much is your monthly income?	……………….ETB	
118	From which source do you get diabetic related health information?	1. Family or friends<br>2. Mass media<br>3. Health professionals<br>4. Diabetes patients<br>5. News letters or leaflets	

		6. Books<br>7. Others specify……………	
109	Do you use internet to find out solution to your disease?	1. Yes<br>2. No	

Part II: Diabetic related clinical information of patients

Instruction: These are questions regarding diabetic related clinical information of patients, please encircle the answer they responded to space provided.

201	Type of diabetes Mellitus	1. Type 1 DM<br>2. Type 2 DM	
202	How long it had been since you were diagnosed with diabetes?	……..years	
203	Do you have Comorbidities?	1. Yes<br>2. No	If no go to Q.204
204	If yes what type of comorbidity do you have?	1. Hypertension<br>2. Nerve problem<br>3. Kidney disease<br>4. Heart problem<br>5. Other(specify)…………….	
205	What treatment regimen currently you are using?	1. Diet and exercise only<br>2. Oral hypoglycemic agent<br>3. Insulin<br>4. Insulin+ oral hypoglycemic agent	
206	Do you have family member having Diabetes Mellitus?	1. Yes<br>2. No<br>3. Not sure	
207	Have you received diabetic education?	1. Yes<br>2. No	
208	Are you member of diabetic association?	1. Yes<br>2. No<br>3. I don’t know about it	

209	How many people are so close to you that you can count on them if you have great personal problems?	1 ‘none’<br>2 ‘1–2’<br>3 ‘3–5’<br>4 ‘5+	
210	How much interest and concern do people show in what you do?	1 ‘none’<br>2 ‘little’<br>3 ‘uncertain’<br>4 ‘some’<br>5 ‘a lot’	
211	How easy is it to get practical help from neighbors if you should need it?	1 ‘very difficult’<br>2 ‘difficult’<br>3 ‘possible’<br>4 ‘easy’<br>5 ‘very easy’	
212	Do you have drink alcohol in the past one year?	1. Yes<br>2. No	If No go to Q215
213	How many times do you consume alcohol?	1.Up to 4 times per month<br>2.More than 4 times per week	
214	Have you smoked cigarette—even one puff—during the past SEVEN DAYS?	1. Yes<br>How many cigarettes did you smoke on an average day?<br>Number of cigarettes:________<br>2. No	

Part III: Questions concerning diabetic health literacy of patients

Instruction: These are questions regarding diabetic health literacy of diabetic patients, please encircle the answer they tell you correctly.

Serial. No.	Questions	Response				
		1. Strongly disagree	2. Disagree	3. Not sure	4. Agree	5. Strongly agree
1. When reading instructions or leaflets from hospitals or pharmacies, how do you agree or disagree about the following?						
401	You find characters that you cannot read	5	4	3	2	1
402	You find that the print was too small to read (even you wear glass)	5	4	3	2	1

403	You feel the content was too difficult to you to understand	5	4	3	2	1
404	It takes to you a long time to read them	5	4	3	2	1
405	you need someone to help you to read them	5	4	3	2	1
2.<br>Since you are diagnosed as having diabetes Mellitus, have you had the following experiences in seeking the information related to diabetes (e.g. diagnosis, treatment, self-care issues, alternative therapy, etc.)?						
406	You collect information from various sources	1	2	3	4	5
407	You extract the information you wanted	1	2	3	4	5
408	You understand the information that you obtained	1	2	3	4	5
409	You tell your opinion about your illness for your doctors, families or friends	1	2	3	4	5
410	you apply the obtained information to your daily life	1	2	3	4	5
3. Since you are diagnosed as having Diabetes Mellitus and you can obtain information about diabetes and its treatment, how do you agree or disagree about the following?						
411	You consider whether the information is applicable to you or not	1	2	3	4	5
412	You consider whether the information is credible	1	2	3	4	5
413	You checked weather the information is valid and reliable	1	2	3	4	5
414	You collect information to make your health care decisions	1	2	3	4	5
