# Supplementary material for: Diabetic health literacy and associated factors among diabetes mellitus patients on follow up at public hospitals, Bale Zone, South East Ethiopia, 2021
Source: PLoS One. 2022 Jul 7;17(7):e0270161. doi: 10.1371/journal.pone.0270161 (PMC9262198; doi:10.1371/journal.pone.0270161)
Supplement: S2 File — (PDF) [file pone.0270161.s002.pdf]

Gaaffii Afaan Oromoo

Kutaa Tokko: Gaaffii Namumaa ilalatu

Qajeelfama 1. Gaafiileen kun waa'ee haawaasumaa ilaallata. maaloo tokko tokkoon waanin isiniif dubiisu sirnaan na caqasaa, sanaan booda deebii ykn yaada sirrii naaff kennaa.

T.Lakk	Gaffii	Deebbii	Darbi
101	Umuriin kee meqaa?	……….	
102	Saala	1. Dhiira<br>2. Dhalaa	
103	Ga'illi kee akkam?	1.Heerumera/ Fudhera<br>2.hin heerumine/ fuune<br>3. kan irraa du'ee<br>4. adda baaneera<br>5. Wal hikneera	
104	Sadarkaan barumsa kee hangam?	1.Baruumsa hin baranne<br>2.kan dubbisuu fi barreesuu danda'u<br>3.Sadarkaa tokkoffaa<br>4.Sadarkaa lamaffaa<br>5.Sadarkaa kolleejii fi isaa ol	
105	Hojjiin kee maalii?	1. Baratаa<br>2. Hojii dhunfaa<br>3. hojjataa mootummaa<br>4. hin qacaramne<br>5. giiftii manaa<br>6.Kan biraa (ibsi)_______	
106	Iddoo jireenyaa	1 .Magaalaa<br>2. Baadiyyaa	
107	Galii Giddu-galeessa maatii qarshiidhaan	qarshii_______	
108	Odeefannoo fayyaa essa arkattu?	1. maatii ykn hiriyoottan irraa<br>2. maas miidiyaa<br>3. oogeysoota fayyaa irraa<br>4. dhukkubsattoota dhibee sukaaraa irrraa<br>5. xalayaalee odeefannoo ykn xalayaa dadeebituu<br>6. kitaaboota<br>7. kan biraa(ibsi)…………….	
109	Dhibee keessaniif furmaata arkachuu intarneetii ni fayyadamtuu?	1. eyyee<br>2. hin fayyadamuu	

Kutaa Lama: Gaaffilee Fayyaa ilaallatu

Qajeelfama 2. Gaafiileen kun haala dhibe sukkaaraa dhibamtoota dhibee sukkaaraa ilaallata. maaloo tokko tokkoon waanin isiniif dubiisu sirnaan na caqasaa, sanaan booda deebii ykn filannoo sirrii naa`ff kennaa.

T.Lakk	Gaaffi	Deebbi	
201	Gosa Dhukkuba Sukkara	1.Gosa tokkoffaa<br>2. Gosa lamaffaa<br>3. hin beeku	
202	Dhibichii waggaa meeqaaf isinirra ture?	Waggaa___________	
203	Dhokkuba dabalata	1.Yes Eeyyee<br>2.No lakki<br>3. don't know Hin beeku	
204	Gaafii lakk. 204 tiif Yoo deebiin kessan Eeyyeen ta'e dhibee kam qabdu?	1.dhibaa dhigaa olka'aa<br>2.rakkoo narvii<br>3.dhukkuba kalee<br>4.rakkoo onnee<br>5. kan biraa(ibsi)<br>_______________________<br>______.	
205	Yeroo amma koricha fayyadamittu	1. marfeedha<br>2. kan liqimifamu<br>3. lamanuu isa<br>4. koricha hin fudhadhu	
206	Maatii kee kessa kan dhibee sukkaaraa dhukubsatte beeku jira	1.Eeyyee<br>2.Lakki<br>3. Hin beeku	
207	Barumsa dhukkuba sukara barate bekta?	1. Gonkuma<br>2. Eeyyee darbee darbee<br>3. Eeyyee yeroo hunda	
208	Miseensa Waldaa dhibee sukkaaraa kessa qabda?	1. Eeyyee<br>2. Lakki<br>3. Waldaan dhibee sukkaaraa jirachu hin beeku	
209	Osoo atii rakkoo cimaa keessa seente namoota hagamiitu siiti dhihoo fi na gargaara jeette itti of abdachuu dandeessaa?	1. Hin jiru<br>2. 1-2<br>3. 3-5<br>4. 5 ol	
210	Naamootni waan ati hojjeettu irratti feedhii fi hubannoon isaan qaban hagami?	1. Hin jiru<br>2. Xiqqaadha<br>3. Murtaa'aa miti<br>4. Murtaa'adha<br>5. Baay'eedha	
211	Gargaarsa qabatamaa ta'ee yoo barbaaddee olla irraa argachuun hagam salphaadha jette yaaddaa?	1. Baay'ee ulfaatadha<br>2. Ullfaatadha<br>3. Ni danda'ama<br>4. Salphaadhaa<br>5. Baay'ee salphaadha	

212	Waggaa tokko darbe kesssatti dhugaatii alkoolii dhugdee beektaa?	1. Eeyye<br>2. Lakkii	Yoo deebiin kee lakkii ta'eera ta'e gara gaafii 217 tti darbi.
213	Yeroo hagamiitiif dhugaatii alkoolii dhugdee ?	1. Ji'a keessaatti hanga yeroo afuriif (4)<br>2. Torbee keessatti yeroo afurii (4) ol	
214	Guyyoota turban darbee keessatti yeroo tokkoofillee yoo ta'e tamboo xuuxxee beektaa?	1. Eeyyee , guyyaa tokko keessatti taboo meeqa xuuxxa(aarsita)?__<br>2. Lakkkii	

Kuutaa saddi:- odeefannoo dandeetti dubbisu, barufii barsiisu wa'ee dhibee sukkaaraa

Qajeelfama 4-gaafin kun dandeetti dubbisu, barufii barsiisuu wa'ee dhibee sukkaaraa, dhukubsattoota dhibee sukkaaraatin deebifamudha! Maaloo akkaata tartiba lakkofsa armaan gadii irratii hundaa'uudhaan, waan an isiniif dubbisu xiyyeefannoon dhageefachuudhaan akkaataa barbaachisummaa isaatiin deebii sirrii ta'ee naaf deebistu.

Tartiba lakkofsaa	gaafii	Deebii filannoo				
		1. Bayyee wali hin galuu	2. wali hin galuu	3. Hin mirkane efannee	4. Wali-gala	5. Bayyee wali-gala
1. Qajeelfamoota ykn xalayaa barartuu hoospitaalootaa ykn dukaanoota qorichaa yeroo duubistan kan armaan gadii kana irratii akkamitti wali-galtu ykn wali-hin galee?						
401	Jechoota ykn quubeewan duubisu hin dandeenyee ni arkituu	5	4	3	2	1
402	Maxansi maxansamee bayyee xixiqqaa ta'ee dubisuuf isiin rakiisee beekaa (fullee ijaa fayyadamtan illee)	5	4	3	2	1
403	Yaadoonii maxansa keessa jiru	5	4	3	2	1

	hubachuuf bayyee ulfaata ta'e akka jiru isinitti dhagahaamaa					
404	Isaan sana dubisudhaaf yeroo dheeraa isinitti fudhataa	5	4	3	2	1
405	Isaan kana dubisuuf nama isiin gargaaruu ni barbaaduu	5	4	3	2	1
**2.**<br>Dhibee sukkaaraa akka qabdan erga qoratamtanii beektan booda, Odeefannoo dhibee sukkaaraa wajiin wal qabatee barbaaduuf kan armaan gadii rawattanii beektuu?(fakkeenyaaf ; qorannoo,yaala,dandeettii of tajaajiluu, filannoo yaalaa fi k.k.f)						
406	Madda addaa addaa irraa ni sasaabduu	1	2	3	4	5
407	Kan sasaabdaan keessa kan barbaaddan ni baastuu	1	2	3	4	5
408	Odeefannoo argattan hubattanirtuu	1	2	3	4	5
409	Waa'ee dhibee keessanii Dooktaroota, maatiifii hiriyoota keessaniif yaada keessan ni qodduufii	1	2	3	4	5
410	Odeefannoo arkattan jireenya guyyaa guyyaa keessan irratti hojittii ni hiktuu	1	2	3	4	5
3. dhuukubsattoonni dhibee suukkaaraa dibicha akka qaban,wa'ee dhibee sukkaaraa fi waa'ee odeefannoo yaalichaa akka argachuu danda'aan akkaataa armaan gadii kana akkamitti wali-galtu ykn wali-hin galee?						
411	Odeefannoon isiniif raawwii ta'uu fi ta'uu dhabuu isaa ilaalcha kessa ni galchituu	1	2	3	4	5
412	Odeefannoon amanamaa ta'uu fi ta'uu dhabuu isaa ilaalcha kessa ni galchituu	1	2	3	4	5
413	Odeefannoon sirriifi maamii kan	1	2	3	4	5

	hin qabnee ta’uu isaa ni mirkaneessituu					
414	Kunuunsa fayyaa kessan murtee goodhachuuf odeefannoo ni sasaabduu	1	2	3	4	5
